# Interplay Between Adherence to the Mediterranean Diet and Lipid Profile: A Comparative Survey Between Day-Time Healthcare and Non-healthcare Female Workers

**DOI:** 10.3389/fpubh.2021.649760

**Published:** 2021-11-04

**Authors:** Luigi Di Lorenzo, Luigi Vimercati, Antonella Pipoli, Nicola Mariano Manghisi, Luisa Lampignano, Antonio Caputi, Luigi De Maria, Roberta Zupo, Giovanni De Pergola

**Affiliations:** ^1^Interdisciplinary Department of Medicine (DIM), Section of Occupational Medicine B. Ramazzini, School of Medicine, University of Bari Aldo Moro, Bari, Italy; ^2^Population Health Unit, Salus in Apulia Study, National Institute of Gastroenterology “Saverio de Bellis” Research Hospital, Bari, Italy; ^3^Department of Biomedical Science and Human Oncology, School of Medicine, University of Bari, Bari, Italy

**Keywords:** Mediterranean diet, blood lipid profile, healthcare workers, health promotion, occupational medicine

## Abstract

**Introduction:** Occupational physicians, as an aspect of the periodic health surveillance of workers prescribed by law, must develop preventive programs against adverse health-related occurrences (Legislative Decree 81/2008, art.25) to reduce major risk factors for non-communicable/chronic diseases. Eating habits play an important role in defining risk trajectories in the workplace.

**Methods:** We randomly and cross-sectionally evaluated 147 females, of which 59 were healthcare workers (HCWs) and 88 were non-HCWs. The assessment included a dietary screening for adherence to the Mediterranean diet (MD) and a clinical baseline collection of major fluid biomarkers and anthropometric indicators for cardiovascular and metabolic risk.

**Results:** The HCW group exhibited greater adherence to the MD than the non-HCW group. Nevertheless, they showed higher serum levels of triglycerides and total cholesterol. Menopause and type of work significantly and unfavorably affected triglyceride serum levels among HCWs.

**Conclusion:** Greater preventive efforts are needed in the context of periodic health surveillance by occupational physicians. Disseminating additional information on a healthier lifestyle, particularly among female workers of perimenopausal age, is a key issue.

## Introduction

The Mediterranean diet (MD) is a diet common to the inhabitants of lands surrounding the Mediterranean Sea, originally observed in Southern Italy in the 1960s and specifically described based on food consumption patterns in the Greek Peninsula (1, 2). This dietary pattern features higher consumption of vegetables and lower intake of animal foods and is widely recognized as a key driver of the lower rate of mortality from cardiovascular disease (CVD), as observed in the Corfu and Crete cohorts at 25 years of follow-up (1). In particular, MD defines a nutritional model characterized by high consumption of seasonal vegetables, fresh fruits and nuts, legumes and cereals, which ensure an appropriate intake of fiber and starch; moreover, a specific peculiarity of MD is the use of extra virgin olive oil (EVOO), rich in monounsaturated fatty acids, as the main lipid source, and low intake of saturated fatty acids. A moderate consumption of fish, dairy products, eggs and red wine (for its antioxidant activity) is allowed (1–4). Several studies have demonstrated the association between adherence to MD and reduced prevalence of cardiovascular risk factors such as abdominal fat (5, 6), hypertension (7), dyslipidemia (8), diabetes mellitus (9), CVD (10, 11) and cancer (12). Interestingly, we found that low adherence to MD was also associated with lower circulating levels of 25-OH-vitamin D (6). All these benefits are largely derived from daily consumption of 25–50 ml/day EVOO, which seems most importantly to work well in decreasing LDL atherogenicity and thus CV risk. This is notable since LDL cholesterol is accepted as a causal risk factor for the development of myocardial infarction and atherosclerotic CVD (13).

Although extensive research has consistently provided proof of the numerous beneficial effects of the MD, especially in the context of prevention of CV and metabolic diseases, a shift of the traditional MD pattern toward the so-called “Western diet” (characterized by a greater consumption of refined grains, sugars and red and processed meats) has paradoxically been occurring over the last few decades, even in Mediterranean countries (14).

These changes in Italian eating habits have resulted from a combination of widespread urbanization, globalization and economic crises, at least since the end of the last century, and are often associated with an increase in sedentary work and lifestyle patterns (15).

Thus, from a preventive perspective, the MD should be promoted to a greater extent in Mediterranean countries, especially among individuals of working age. In this regard, the role of occupational physicians is of particular significance, as these professionals are responsible for the management and prevention of diseases related to workplace factors, especially to ensure regular health surveillance of workers exposed to occupational health and safety risks (16). In addition, in high-income countries, health promotion is useful for balancing the reduction of work-related metabolic and CV risk factors with the increase in subjective risk factors due to new and often unhealthy lifestyles (17). The practice of using lifestyle corrective programs during periodic worker health surveillance by occupational physicians may be exceptionally useful in reducing the risk of chronic diseases, particularly in southern Italy, where, at the expense of a higher incidence of obesity, there is a lower prevalence of obesity treatment centers (18, 19).

Indeed, several Italian studies have shown that the promotion of MD in the workplace helps to increase the consumption of whole grains, legumes, white meat and fish and to moderate that of foods rich in cholesterol, saturated fats and sugars (20). In a sample of Italian healthcare workers (HCWs), it was found that the adoption of unhealthy lifestyles, together with shift work, is an additional risk factor for CV and metabolic diseases (21). Accordingly, a recent record from US HCWs showed that greater adherence to MD can reduce CV risk factors and the incidence of coronary heart disease (CHD) (22). This result is of particular interest because HCWs show higher CV risk due to the higher number of work-related stressors (23). Finally, it should be noted that CV and metabolic risk increase significantly with menopause, and this physiological condition may accompany 15–20 years of female working age (24).

The objective of this study was to assess possible differences in adherence to the MD and major metabolic and anthropometric parameters between adult female HCWs (healthcare workers) and non-HCWs (non-Healthcare Workers), to determine any association that could help occupational physicians improve preventive health risk management.

## Materials and Methods

### Study Population and Design

A transversal observational study was conducted. One hundred forty-seven subjects were randomly recruited at the Operating Unit of Occupational Medicine of the University Polyclinic Hospital of Bari (Apulia, Southern Italy) in the last quarter of 2019. The whole sample included only female employees of public companies with at least 16 years of education and subject to health surveillance by the occupational physicians of the above operating unit, subdivided as follows: 59 HCWs (doctors and professional nurses employed at the University Hospital of Bari) and 88 employees of other public companies not operating in the field of healthcare (non-HCWs). All the workers were Italian and lived in Puglia. Given that the working schedule of companies employing non-HCWs did not include both day and night shifts, only non-shift HCWs were recruited to avoid the influence of shift work on eating habits and metabolic status (25). Biological sex differentially determines susceptibility to cardiovascular and metabolic risks, so we preferred to analyze only female workers in this study (26).

The study protocol (ClinicalTrials.gov Identifier: NCT04596358) met the principles of the Declaration of Helsinki and was approved by the Ethics Committee of the National Cancer Research Center “Giovanni Paolo II,” Bari, Italy. All participants gave informed consent prior to enrollment, in accordance with the Helsinki Declaration of 1964 and subsequent revisions.

### Clinical Examination and Fluid Biomarker Collection

As part of the periodic health surveillance, a senior physician conducted a cross-sectional evaluation consisting of a brief interview on medical history and lifestyle. Extemporaneous outpatient diastolic blood pressure (DBP) and systolic blood pressure (SBP) were determined in a sitting position after at least a 10-min rest, a minimum of three different times, using an OMRON M6 automatic blood pressure monitor. Subjects with BP values > 130/85 mm Hg or already under drug therapy were classified as currently suffering from hypertension (27).

Metabolic and routine biochemical parameters were closely examined in all subjects. Blood samples were drawn between 08:00 and 09:00 a.m. after overnight fasting. Fasting plasma glucose (FPG), total cholesterol, high- and low-density lipoprotein (HDL, LDL) cholesterol, and triglyceride (TG) serum levels were assayed. Plasma glucose was determined using the glucose oxidase method (Sclavus, Siena, Italy), while the concentrations of plasma lipids (triglycerides, total cholesterol, HDL cholesterol) were quantified by the automated colorimetric method (Hitachi; Boehringer Mannheim, Mannheim, Germany). LDL cholesterol was directly determined in blood samples. Subjects with circulating LDL cholesterol > 116 mg/dl or already under drug therapy were classified as having hypercholesterolemia (28).

### Anthropometric Assessment

Clinical procedures were performed by two qualified nutritionists (RZ and LL) trained for equivalent measuring performances. All anthropometric measurements were taken with participants dressed in lightweight clothing and without shoes. Variables were all collected at the same time between 7:00 and 10:00 a.m. after overnight fasting. Height was measured to the nearest 0.5 cm using a wall-mounted stadiometer (Seca 711; Seca, Hamburg, Germany). Body weight was determined to the nearest 0.1 kg using a calibrated balance beam scale (Seca 711; Seca, Hamburg, Germany). BMI was calculated by dividing body weight (kg) by the square of height (m^2^) and classified according to World Health Organization criteria for normal weight (18.5–24.9 kg/m^2^), overweight (25.0–29.9 kg/m^2^), grade I obesity (30.0–34.9 kg/m^2^), grade II obesity (35.0–39.9 kg/m^2^), and grade III obesity (≥40.0 kg/m^2^) (29). Waist circumference (WC) was measured at the narrowest part of the abdomen or in the area between the 10th rib and the iliac crest (minimum circumference). Subjects with previous diagnoses of hypertension, diabetes mellitus, hypercholesterolemia, hypertriglyceridemia and related drug therapies were identified. In addition, subjects with physiological amenorrhea for at least 12 months, as well as those with iatrogenic amenorrhea, were recorded as menopausal (30).

### Assessment of Adherence to a MD

Two senior nutritionists (RZ and LL) administered a previously validated 11-item index, the MedDietScore (MD score) (31), to assess adherence to a MD. The MD score developed by Panagiotakos and colleagues was chosen because of its corroborated applicability and psychometric quality (32). Female workers were interviewed individually at the time of medical examination by the above nutritionists.

For food items presumed to be close to the MD pattern (i.e., those that are suggested to be consumed on a daily basis or in >4 servings/week: unrefined cereals, fruits, vegetables, potatoes, legumes, olive oil and fish), a score of 0 was assigned when a participant reported no consumption, a score of 1 was assigned for reported consumption of 1–4 times/month, a score of 2 for 5–8 times/month, a score of 3 for 9–12 times/month, a score of 4 for 13–18 times/month and a score of 5 for >18 times/month. In contrast, for the consumption of foods presumed to be inconsistent with this dietary pattern (i.e., those suggested not to be consumed on a daily or weekly basis: meat and meat products, poultry and high-fat dairy products), the opposite scores were assigned (i.e., a score of 0 when a participant reported almost daily consumption of the food to a score of 5 for rare or no consumption). For alcohol consumption, a non-monotonic scoring was adopted based on daily intake of 15–30 g ethanol as suggested by the MD pattern (i.e., a score of 5 was assigned for consumption of <3 glasses/d; 0 for none or consumption of >7 glasses/d; and scores of 4, 3, 2 and 1 for the consumption of 3, 4–5, 6 and 7 glasses/d, respectively). The resulting total score ranged from 0 to 55.

### Statistical Analysis

Data are reported as the mean ± standard deviation (M ± SD) for continuous measures and frequency and percentage (%) for all categorical variables. The normality of distribution was assessed for each variable using Shapiro's test. Comparisons of the means of the continuous variables between HCWs and non-HCWs were performed with Student's *t*-test for independent samples. Comparisons of the means of the continuous variables of the stratified women workers based on menopause were performed with ANOVA. Comparisons of the categorical variables were performed with the χ^2^ test. The averages of total cholesterol, LDL, HDL, TG, SBP and DBP were calculated only for subjects who were not on therapy for hypertension or hypercholesterolemia.

Correlation tests were performed with Pearson's test. A multivariate logistic regression test was performed to calculate the odds ratio (OR). UNIANOVA univariate linear analysis models or two-way variance tests permitted evaluation of the effect of two combined experimental factors to highlight possible interaction effects. By interaction effect, we mean the phenomenon whereby the effect of one factor changes depending on the level of the other factor. A *p*-value ≤ 0.05 was considered statistically significant, with a 95% confidence interval, and *p*-values not statistically significant are shown in the tables as “n.s.” All analyses were performed using SPSS Statistics Software.

## Results

The whole sample (*N* = 147) featured a majority of non-HCWs (*N* = 88). Table 1 summarizes the general, anthropometric and metabolic laboratory parameters of the enrolled subjects, expressed as the mean ± SD, for continuous variables and as percentages (%) for proportions. Age, WC and BMI averages were not significantly different between the two groups. HCWs showed significantly greater MD scores and total cholesterol and TG serum levels but no significant differences in other clinical variables measured [HDL- and LDL-cholesterol serum levels, fasting blood glucose, diastolic and systolic blood pressure (DBP, SBP)]. Regarding each group of foods, HCWs showed higher intake of potatoes, legumes and fish (*p* < 0.001) than non-HCWs. Additionally, HCWs consumed significantly higher quantities of red meat and its derivatives, white meat (poultry) and alcoholic beverages (*p* < 0.001) (Table 2).

**Table 1:** Descriptive statistics for the whole sample subdivided by HCWs and non-HCWs.

	**HCWs**		**Non-HCWs**			
**Variable**	**59**		**88**			
	**Mean**	**SD**	**Mean**	**SD**	**t-test**	**p**
Age (years)	46.0	(8.9)	44.0	(10.0)		n.s.
BMI (kg/m^2^)	24.0	(5.4)	24.5	(4.5)		n.s.
WC (cm)	86.8	(11.2)	83.7	(10.7)		n.s.
MD Score	35.8	(4.7)	34.1	(3.7)	2.3	<0.05
Total cholesterol (mg/dl)^a^	191.6	(32.8)	180.5	(25.8)	2.3	<0.05
HDL-cholesterol (mg/dl)^a^	66.3	(17.5)	66.4	(15.3)		n.s.
LDL-cholesterol (mg/dl)^a^	107.1	(31.1)	109.8	(29.4)		n.s.
TG (mg/dl)	94.7	(44.8)	75.7	(31.4)	2.8	<0.05
FBG (mg/dl)	81.5	(9.4)	84.4	(11.3)		n.s.
SBP (mmHg)^b^	114.2	(16.0)	110.9	(14.2)		n.s.
DBP (mmHg)^b^	72.4	(9.7)	71.6	(10.2)		n.s.

a *The averages for total cholesterol, LDL and HDL Were calculated only considering subjects who were not on therapy for hypercholesterolemia*.

b *The averages for SBP and DBP were calculated only for subjects who were not on therapy for hypertension*.

*BMI, body mass index; WC, waist circumference; TG, triglycerides; FBG, fasting blood glucose; SBP, systolic blood pressure; DBP, diastolic blood pressure; n.s., not significant*.

**Table 2:** Comparison between the mean score for each food category included in the Mediterranean diet in the HCW and non-HCW groups.

	**HCWs**		**Non-HCWs**			
**Score**	**Mean**	**SD**	**Mean**	**SD**	**t-test**	**p**
Cereals	4.5	(0.5)	4.7	(0.8)		n.s.
Potatoes	3.1	(1.6)	1.0	(1.1)	8.7	<0.001
Fruits	4.6	(0.9)	4.8	(0.7)		n.s.
Vegetables	4.6	(0.9)	4.6	(0.8)		n.s.
Legumes	3.5	(1.6)	1.8	(1.0)	7.3	<0.001
Fish	3.8	(1.3)	1.5	(0.9)	11.9	<0.001
Red meat products and eggs^*^	1.1	(1.4)	2.0	(1.4)	−3.7	<0.001
Poultry^*^	1.3	(1.4)	3.3	(1.0)	−9.5	<0.001
Cheese. yogurt and milk^*^	0.7	(1.4)	0.7	(1.5)		n.s.
EVOO	4.6	(0.9)	4.6	(0.9)		n.s.
Alcohol^*^	3.9	(1.7)	4.8	(0.7)	−3.7	<0.001

* *lower scores correspond to more frequent consumption, considered less adherent to the Mediterranean diet*.

*EVOO, extra-virgin olive oil; n.s., not significant*.

The correlation analysis matrix for the whole sample (Table 3) showed significant inverse associations between the MD score and LDL cholesterol (*p* < 0.05) and between EVOO consumption and LDL cholesterol (*p* < 0.01). Significant positive correlations were found between TG serum level and age, BMI, and WC (*p* < 0.001). The same patterns were also found for LDL cholesterol, fasting blood glucose, DBP and SBP.

**Table 3:** Correlation matrix for continuous variables across the entire study population.

	**HCWs** **+** **Non-HCWs (*****n*** **=** **147)**									
	**TG**		**LDL-cholesterol**		**FBG**		**SBP**		**DBP**	
	**(mg/dl)**		**(mg/dl)**		**(mg/dl)**		**(mmHg)**		**(mmHg)**	
	**r**	**p**	**r**	**p**	**r**	**p**	**r**	**p**	**r**	**p**
Age (years)	0.27	<0.001	0.28	<0.01	0.21	<0.01	0.43	<0.001	0.34	<0.001
BMI (kg/m^2^)	0.28	<0.001	0.15	<0.05	0.25	<0.001	0.33	<0.001	0.36	<0.001
WC (cm)	0.34	<0.001	0.17	<0.05	0.23	<0.05	0.33	<0.001	0.33	<0.001
EVOO Score	−0.01	n.s.	−0.20	<0.01	−0.12	n.s.	−0.02	n.s.	−0.10	n.s.
MD Score	0.03	n.s.	−0.18	<0.05	−0.05	n.s.	0.06	n.s.	−0.04	n.s.

*BMI, body mass index; WC, waist circumference; EVOO, extra-virgin olive oil; MD Score, Mediterranean diet score; TG, triglycerides; FBG, fasting blood glucose; SBP, systolic blood pressure; DBP, diastolic blood pressure; n.s., not significant*.

In the multivariate logistic regression model, LDL cholesterol level > 116 mg/dl was set as the dependent variable, and work category, MD Score > 35, EVOO consumption >/= 5 servings per week (1 serving = 25 ml) were set as the independent variables. The above model showed that LDL cholesterol levels were positively influenced by menopausal status (OR = 2.264, 95% CI 1.045–4.907; *p* = 0.038) and negatively influenced by EVOO consumption (OR = 0.280, 95% CI 0.104–0.759; *p* = 0.012).

Differences in categorical variables between the two groups are shown in Table 4. The HCW group included greater numbers of subjects with menopausal status, with hypertension and with hypertriglyceridemia (*p* < 0.05) than the non-HCW group.

**Table 4:** Comparison of categorical variables between the HCW and non-HCW groups.

	**HCWs (*****n*** **=** **59)**		**Non-HCWs (*****n*** **=** **88)**			
	**n**	**%**	**n**.	**%**	**χ^2^**	**p**
Menopause	22	(37)	20	(23)	0.95	<0.05
LDL-cholesterol > 116 mg/dl^a^	21	(36)	28	(32)	0.2	n.s.
Treatment for hypercholesteremia	7	(12)	5	(6)	3.0	n.s.
SBP >130 mm Hg and/or DBP >85 mm Hg^b^	6	(10)	3	(3)	2.9	n.s.
Treatment for hypertension	8	(14)	3	(3)	5.9	<0.05
TG > 150 mg/dl	9	(15)	4	(5)	4.8	<0.05

a *Subjects under treatment for hypercholesterolemia were not included*.

b *Subjects under treatment for hypertension were not included*.

*SBP, systolic blood pressure; DBP, diastolic blood pressure; TG, triglycerides; n.s., not significant*.

UNIANOVA analysis was used to assess whether the type of work (healthcare or non-healthcare) or menopausal status could have any effect, singly or in combination, on SBP, DBP, circulating LDL cholesterol, TG serum levels, and fasting blood glucose. We found that menopause had a significant effect on SBP, DBP, LDL cholesterol and blood sugar, regardless of the type of work performed. Higher TG serum levels in HCWs were found to be affected both by menopausal status and type of job (Table 5).

**Table 5:** UNIANOVA for the analysis of variance in fluid biomarkers between the two groups (HCWs and non-HCWs), further subdivided according to menopausal status, and evaluation of the single or combined effects of job and menopause as confounders.

**Job**	**HCWs (*****n*** **=** **59)**				**Non-HCWs (*****n*** **=** **88)**									
**Menopause**	**Yes (*****n*** **=** **22)**		**No (*****n*** **=** **37)**		**Yes (*****n*** **=** **20)**		**No (*****n*** **=** **68)**		**Menopause effect**		**Job effect**		Job* **Menopause effect**	
	**M**	**S.D**.	**M**	**S.D**.	**M**	**S.D**.	**M**	**S.D**.	**F**	**p**	**F**	**p**	**F**	**p**
SBP (mm Hg)^b^	121.3	16.5	111.4	13.9	121.6	10.1	108.0	13.8	25.6	<0.01		n.s.		n.s.
DBP (mm Hg)^b^	73.4	11.3	71.9	8.5	76.7	6.4	70.2	10.4	4.3	<0.05		n.s.		n.s.
LDL cholesterol (mg/dl)^a^	125.3	25.5	98.3	32.5	124.7	41.6	106	24.8	14.0	<0.01		n.s.		n.s
TG (mg/dl)	118.2	48.0	77.7	33.2	81.7	32.7	71.6	28.5	16.3	<0.01	11.6	<0.01	5.9	<0.05
FBG (mg/dl)	87.1	9.8	78.2	7.4	87.9	15.1	83.4	10.0	12.2	<0.01		n.s.		n.s.

a *The averages for total cholesterol, LDL and HDL were calculated only considering subjects who were not being treated for hypercholesterolemia*.

b *The averages for SBP and DBP were calculated only for subjects who were not being treated for hypertension*.

*TG, triglycerides; FBG, fasting blood glucose; SBP, systolic blood pressure; DBP, diastolic blood pressure; n.s., not significant*.

## Discussion

This study shows that HCWs are more MD-adhering than are non-HCWs, as demonstrated by the higher average MD score, implying protection against major non-communicable diseases, perhaps due to their professional knowledge. Despite being arithmetically close, the two averages were significantly different (mean difference 1.7, CI 95% −3.2 to −0.4, *p* = 0.012), likely due to the limited dispersion of MD score values relative to the average, as documented by the relatively low standard deviations in both groups.

Surprisingly, HCWs showed significantly higher total cholesterol and triglyceride serum levels, likely related to their higher consumption of red meat and its derivatives, eggs, and alcoholic beverages, all foods capable of interfering with lipid metabolism and, probably, of counteracting the beneficial effects of foods more adherent to the MD.

The higher alcohol consumption found in the HCW group opens a very interesting research area with the intent of verifying the hypothesis of alcohol use as a potential sensitive indicator of the level of work-related stress (33), a topic that does not actually fall within our main goals.

Corroborating the internal validity of our study, subjects with higher age, BMI and abdominal circumference (WC) exhibited higher circulating TG, LDL cholesterol, fasting blood glucose serum level, and blood pressure. Indeed, it is well-known that age, excess weight and visceral adiposity can contribute to increase risks of hypertriglyceridemia, hypercholesterolemia, diabetes and hypertension (34). In addition, our study confirms that even in Mediterranean countries, especially in southern Italy, where EVOO consumption is a common everyday practice, there is better control of LDL cholesterol levels in subjects who follow this custom more closely (35, 36).

Furthermore, the HCW group, including a larger number of subjects in menopause, stands out for higher levels of TG. In this regard, the multivariate logistic regression model and the analysis of two-way variance highlighted that the condition of menopause exerts strong effects on all metabolic fluid biomarkers investigated. In particular, our analyses reveal that menopausal status has a significant adverse effect on TG levels among HCWs. This finding confirms previous evidence supporting the capability of menopause to induce, especially during the transition period, significant metabolic changes that may persist even in late life. Elevated blood lipids, especially LDL cholesterol serum levels, have been recently recorded in both cross-sectional and longitudinal settings (37). In addition, the lack of estrogen and progesterone, characteristic of menopause, contributes to an increase in blood pressure and an increase in overall cardiovascular risk (38).

Some limitations of this study should be considered. Because of the cross-sectional approach, it has not been possible to establish the direction of any causal relationship, so our data provide a description rather than an explanation. Prospective studies are needed to clarify causal relationships. In addition, we failed to collect further data on other determinants that may influence the eating habits and metabolic status of female workers, such as marital status, number of children, physical activity, and work-related stress. It would also be interesting to assess shift workers to test whether their eating habits and lifestyle are influenced by work organization and, above all, by night work.

Finally, the choice of defining the state of menopause in the anamnestic phase as authoritatively proposed recently by Nelson (30), without hormonal dosage, may have compromised the methodological accuracy. However, this choice was driven by the intent of facilitating the application of this health-promotion protocol during the periodic health surveillance of workers, avoiding proposing the administration of hormonal doses that may influence the sensitivity of workers, as well as the requirement of more complex and therefore fairly expensive laboratory techniques.

It also should be noted that, although the limited sample size, the study subjects were selected in such a way that the two groups were as homogeneous as possible. In particular, the two groups of female workers were homogeneous in terms of various characteristics capable of influencing metabolic status and lifestyles (age, level of education, WC, BMI). Therefore, HCW and non-HCW groups are adequately comparable to ensure a proper statistical analysis of the results. Moreover, according to the formula proposed by Viechtbauer et al., a minimum sample size of 58 subjects is required to perform pilot studies using a 95% confidence interval. The sample under study consisting of 147 female workers, therefore, should be adequate to perform a proper statistical analysis (39) however we propose to enlarge the sample for further studies.

## Conclusions And Pratical Implications

HCWs are more adherent to a MD than are non-HCWs, possibly due to greater knowledge of healthy principles related to this dietary pattern. Despite this, HCWs showed higher serum concentrations of triglycerides and total cholesterol. Both menopausal status and type of work were linked to higher TG levels among HCWs.

According to the results of the study, the main area of intervention is represented by periodic health surveillance in which the occupational physician will have to propose to the female workers clear and personalized indications on the qualitative and quantitative composition of the diet, also in consideration of the energy expenditure required to carry out the specific tasks.

Periodic health surveillance is also an opportunity to better investigate alcohol consumption and look for possible associations with work-related stress factors. This topic could be the subject of future studies.

Recent scientific evidence confirms the importance of gender medicine also in the field of occupational medicine. In fact, our results show that the menopause has significant effects on the metabolism and cardiovascular system in women. It is therefore necessary for occupational physicians to develop health promotion programmes specifically aimed at women to counteract the menopausal effects on their health.

In addition, to help female workers correct their blood levels of TG and Total Cholesterol, public institutions and private companies should promote their physical activity by entering into agreements with gyms or sports centers and, where possible, by creating jogging and/or walking routes within the company perimeter or by providing suitable company premises with sports equipment (e.g., treadmills and/or exercise bikes). In addition, employers of companies with workplace canteens should require catering companies to provide nutritionally balanced menus, indicating the calories and nutrients in each dish. Another useful intervention would be to introduce low-calorie drinks and snacks in vending machines that are free or low in refined sugars and/or saturated fats and/or salt, respectively. All these interventions should be agreed with the occupational physician and shared with the workers' safety representatives.

## Data Availability Statement

The raw data supporting the conclusions of this article will be made available by the authors, without undue reservation.

## Ethics Statement

The studies involving human participants were reviewed and approved by Ethics Committee of the National Cancer Research Center Giovanni Paolo II, Bari, Italy (ClinicalTrials.gov, Identifier: NCT04596358). The patients/participants provided their written informed consent to participate in this study.

## Author Contributions

LDi: conceptualization, visualization, and project administration. AP and LV: methodology. AP: software and formal analysis. GD and LV: validation. NM and LL: investigation. GD: resources and supervision. AP, AC, and LDe: data curation. RZ, AP, AC, and LDe: writing—original draft preparation. LDi and GD: writing—review and editing. All authors have read and agreed to the published version of the manuscript.

## Conflict of Interest

The authors declare that the research was conducted in the absence of any commercial or financial relationships that could be construed as a potential conflict of interest.

## Publisher's Note

All claims expressed in this article are solely those of the authors and do not necessarily represent those of their affiliated organizations, or those of the publisher, the editors and the reviewers. Any product that may be evaluated in this article, or claim that may be made by its manufacturer, is not guaranteed or endorsed by the publisher.

## References

[1] Menotti A Kromhout D Blackburn H Fidanza F Buzina R Nissinen A. Food intake patterns and 25-year mortality from coronary heart disease: cross-cultural correlations in the seven countries study. Eur J Epidemiol. (1999) 15:507–15. 10.1023/A:1007529206050 10485342

[2] Fidanza F. Who remembers the true Italian Mediterranean diet? Diabetes Nutr Metab. (2001) 14:119–20. 11476357

[3] Alberti-Fidanza A Fidanza F Chiuchiù MP Verducci G Fruttini D. Dietary studies on two rural Italian population groups of the seven countries study. 3. Trend of food and nutrient intake from 1960 to 1991. Eur J Clin Nutr. (1999) 53:854–60. 10.1038/sj.ejcn.1600865 10556997

[4] D'Alessandro A De Pergola G. Mediterranean diet pyramid: a proposal for Italian people. Nutrients. (2014) 6:4302–16. 10.3390/nu6104302 25325250 PMC4210917

[5] De Pergola G Martino T Zupo R Caccavo D Pecorella C Paradiso S. 25 Hydroxyvitamin D levels are negatively and independently associated with fat mass in a cohort of healthy overweight and obese subjects. Endocr Metabol Immun Disord Drug Targets. (2019) 19:838–44. 10.2174/1871530319666190122094039 30666920 PMC7360908

[6] Zupo R Lampignano L Lattanzio A Mariano F Osella AR Bonfiglio C. Association between adherence to the Mediterranean diet and circulating vitamin D levels. Int J Food Sci Nutr. (2020) 71:1–7. 10.1080/09637486.2020.1744533 32223463

[7] De Pergola G D'Alessandro A. Influence of Mediterranean diet on blood pressure. Nutrients. (2018) 10:1170. 10.3390/nu10111700 30405063 PMC6266047

[8] Schwingshackl L Krause M Schmucker C Hoffmann G Rücker G Meerpohl JJ. Impact of different types of olive oil on cardiovascular risk factors: a systematic review and network meta-analysis. Nutr Metabol Cardiovasc Dis. (2019) 29:1030–39. 10.1016/j.numecd.2019.07.001 31378629

[9] Uusitupa M Khan TA Viguiliouk E Kahleova H Rivellese AA Hermansen K. Prevention of type 2 diabetes by lifestyle changes: a systematic review and meta-analysis. Nutrients. (2019) 11:2611. 10.3390/nu11112611 31683759 PMC6893436

[10] D'Alessandro A De Pergola G. Mediterranean diet and cardiovascular disease: a critical evaluation of a priori dietary indexes. Nutrients. (2015) 7:7863–88. 10.3390/nu7095367 26389950 PMC4586562

[11] Salas-Salvadó J Becerra-Tomás N García-Gavilán JF Bulló M Barrubés L. Mediterranean diet and cardiovascular disease prevention: what do we know? Progr Cardiovasc Dis. (2018) 61:62–7. 10.1016/j.pcad.2018.04.006 29678447

[12] D'Alessandro A De Pergola G Silvestris F. Mediterranean diet and cancer risk: an open issue. Int J Food Sci Nutr. (2016) 67:593–605. 10.1080/09637486.2016.1191444 27251477

[13] Hernáez Á Castañer O Goday A Ros E Pintó X Estruch R. The Mediterranean diet decreases LDL atherogenicity in high cardiovascular risk individuals: a randomized controlled trial. Mol Nutr Food Res. (2017) 61:1601015. 10.1002/mnfr.201601015 28371298

[14] Moreno LA Sarría A Popkin BM. The nutrition transition in Spain: a European Mediterranean country. Eur J Clin Nutr. (2002) 56:992–1003. 10.1038/sj.ejcn.1601414 12373620

[15] Genin PM Dessenne P Finaud J Pereira B Dutheil F Thivel D. Effect of work-related sedentary time on overall health profile in active vs. inactive office workers. Front Public Health. (2018) 6:279. 10.3389/fpubh.2018.00279 30327763 PMC6174317

[16] Scapellato ML Comiati V Buja A Buttignol G Valentini R Burati V. Combined before-and-after workplace intervention to promote healthy lifestyles in healthcare workers (STI-VI study): short-term assessment. Int J Environ Res Public Health. (2018) 15:2053. 10.3390/ijerph15092053 30235849 PMC6164287

[17] Di Lorenzo L Pipoli A Manghisi NM Clodoveo ML Corbo F De Pergola G. Nutritional hazard analysis and critical control points at work (NACCPW): interdisciplinary assessment of subjective and metabolic work-related risk of the workers and their prevention. Int J Food Sci Nutr. (2020) 71:1–7. 10.1080/09637486.2020.1750572 32255377

[18] Watanabe M Risi R De Giorgi F Tuccinardi D Mariani S Basciani S. Obesity treatment within the Italian national healthcare system tertiary care centers: what can we learn? Eat Weight Disord. (2020) 26:771–8. 10.1007/s40519-020-00936-1 32451949

[19] Bonaccio M Di Castelnuovo A Bonanni A Costanzo S De Lucia F Persichillo M. Decline of the Mediterranean diet at a time of economic crisis. Results from the moli-sani study. Nutr Metabol Cardiovasc Dis. (2014) 24:853–60. 10.1016/j.numecd.2014.02.014 24819818

[20] Vitale M Bianchi MA Rapetti V Pepe JM Giacco A Giacco R. A nutritional intervention programme at a worksite canteen to promote a healthful lifestyle inspired by the traditional Mediterranean diet. Int J Food Sci Nutr. (2018) 69:117–24. 10.1080/09637486.2017.1336515 28610484

[21] Pietroiusti A Neri A Somma G Coppeta L Iavicoli I Bergamaschi A. Incidence of metabolic syndrome among night-shift healthcare workers. Occup Environ Med. (2010) 67:54–7. 10.1136/oem.2009.046797 19737731

[22] Yu E Rimm E Qi L Rexrode K Albert CM Sun Q. Diet, lifestyle, biomarkers, genetic factors, and risk of cardiovascular disease in the nurses' health studies. Am J Public Health. (2016) 106:1616–23. 10.2105/AJPH.2016.303316 27459449 PMC4981798

[23] Juárez-Pérez CA Aguilar-Madrid G Haro-García LC Gopar-Nieto R Cabello-López A Jiménez-Ramírez C. Increased cardiovascular risk using atherogenic index measurement among healthcare workers. Arch Med Res. (2015) 46:233–9. 10.1016/j.arcmed.2015.03.002 25797688

[24] Sasser AC Rousculp MD Birnbaum HG Oster EF Lufkin E Mallet D. Economic burden of osteoporosis, breast cancer, and cardiovascular disease among postmenopausal women in an employed population. Womens Health Issues. (2005) 15:97–108. 10.1016/j.whi.2004.11.006 15894195

[25] Ulhôa MA Marqueze EC Burgos LGA Moreno CRC. Shift work and endocrine disorders. Int J Endocrinol. (2015) 2015:826249. 10.1155/2015/826249 25892993 PMC4393906

[26] O'Neil A Scovelle AJ Milner AJ Kavanagh A. Gender/sex as a social determinant of cardiovascular risk. Circulation. (2018) 137:854–64. 10.1161/CIRCULATIONAHA.117.028595 29459471

[27] Whelton PL Carey RM Aronow WS Casey DE Collins KJ Dennison Himmelfarb C. 2017 ACC/AHA/AAPA/ABC/ACPM/AGS/APhA/ASH/ASPC/NMA/PCNA guideline for the prevention, detection, evaluation, and management of high blood pressure in adults: a report of the American College of Cardiology/American Heart Association Task Force on clinical practice guidelines. J Am Coll Cardiol. (2018) 71:e127–248. 29146535 10.1016/j.jacc.2017.11.006

[28] Arnett DK Blumenthal RS Albert MA Buroker AB Goldberger ZD Hahn EJ. 2019 ACC/AHA guideline on the primary prevention of cardiovascular disease: executive summary: a report of the American College of Cardiology/American Heart Association task force on clinical practice guidelines. Circulation. (2019) 140:e563–95. 10.1161/CIR.0000000000000677 30879339 PMC8351755

[29] Ulijaszek SJ. Obesity: preventing and managing the global epidemic. Report of a WHO consultation. WHO technical report series 894. Pp. 252. (World Health Organization, Geneva, 2000.) SFr 56.00, ISBN 92-4-120894-5, paperback. J Biosoc Sci. (2003) 35:624–5. 10.1017/S0021932003245508 11234459

[30] Nelson HD. Menopause. Lancet. (2008) 371:760–70. 10.1016/S0140-6736(08)60346-3 18313505

[31] Panagiotakos DB Polystipioti A Papairakleous N Polychronopoulos E. Long-term adoption of a Mediterranean diet is associated with a better health status in elderly people; a cross-sectional survey in Cyprus. Asia Pac J Clin Nutr. (2007) 16:331–7. 17468091

[32] Zaragoza-Martí A Cabañero-Martínez MJ Hurtado-Sánchez JA Laguna-Pérez A Ferrer-Cascales R. Evaluation of Mediterranean diet adherence scores: a systematic review. BMJ Open. (2018) 8:e019033. 10.1136/bmjopen-2017-019033 29478018 PMC5855302

[33] Ruotsalainen JH Verbeek JH Mariné A Serra C. Preventing occupational stress in healthcare workers. Cochrane Database Syst Rev. (2015) CD002892. 10.1002/14651858.CD002892.pub5 25847433 PMC6718215

[34] Consultation WHO. Obesitiy: Preventing and Managing the Global Epidemic. Washington, DC: World Health Organization. (2000). 11234459

[35] Franconi F Campesi I Romani A. Is extra virgin olive oil an ally for women's and men's cardiovascular health? Cardiovasc Ther. (2020) 2020:6719301. 10.1155/2020/6719301 32454893 PMC7212338

[36] Tsartsou E Proutsos N Castanas E Kampa M. Network meta-analysis of metabolic effects of olive-oil in humans shows the importance of olive oil consumption with moderate polyphenol levels as part of the Mediterranean diet. Front Nutr. (2019) 6:6. 10.3389/fnut.2019.00006 30809527 PMC6379345

[37] Wang Q Ferreira DLS Nelson SM Sattar N Ala-Korpela M Lawlor DA. Metabolic characterization of menopause: cross-sectional and longitudinal evidence. BMC Med. (2018) 16:17. 10.1186/s12916-018-1008-8 29402284 PMC5800033

[38] Mattioli AV Sciomer S Moscucci F Maiello M Cugusi L Gallina S. Cardiovascular prevention in women: a narrative review from the Italian society of cardiology working groups on “cardiovascular prevention, hypertension and peripheral circulation” and on “women disease.” J Cardiovasc Med. (2019) 20:575–83. 10.2459/JCM.0000000000000831 31246698

[39] Viechtbauer W Smits L Kotz D Budé L Spigt M Serroyen J. A simple formula for the calculation of sample size in pilot studies. J Clin Epidemiol. (2015) 68:1375–9. 10.1016/j.jclinepi.2015.04.014 26146089

